# Cunermuspir, a Copper(I)–Niacin Complex, Modulates Mitochondrial Respiration and Cellular Oxidant Handling in Fibroblasts from Children with Autism Spectrum Disorder

**DOI:** 10.3390/antiox15091147

**Published:** 2026-09-10

**Authors:** Sophie Wallace, Spencer Lawes, Adrienne C. Scheck, Richard E. Frye

**Affiliations:** 1 Autism Discovery and Treatment Foundation, Phoenix, AZ 85024, USA; swallace@autismtx.org (S.W.); spencer@autismtx.org (S.L.); adrienne@autismtx.org (A.C.S.); 2 Department of Basic Medical Sciences, University of Arizona College of Medicine—Phoenix, Phoenix, AZ 85004, USA; 3 Rossignol Medical Center, Phoenix, AZ 85018, USA

**Keywords:** copper, cuproenzyme, cytochrome c oxidase, Complex IV, mitochondrial respiration, proton leak, reactive oxygen species, redox modulation, autism spectrum disorder, Cunermuspir

## Abstract

Copper is a redox-active transition metal that is essential for the assembly and catalytic function of cytochrome c oxidase (Complex IV), the terminal enzyme of the mitochondrial electron transport chain and a principal site of physiological oxygen reduction. Elevated Complex IV activity and respiratory chain uncoupling are among the most consistently replicated biological findings in autism spectrum disorder (ASD), yet the interaction between mitochondrial copper delivery, respiration, and cellular oxidant handling in ASD has not been systematically defined. We treated dermal fibroblasts from 9 children with ASD and 10 typically developing controls with the copper(I)–niacin complex Cunermuspir (0, 50, or 100 µM for 1 or 24 h exposure) and challenged them with graded concentrations (0–5.0 µM) of the redox-cycling agent 2,3-dimethoxy-1,4-naphthoquinone (DMNQ). Mitochondrial respiration was profiled by Seahorse XF respirometry (2133 observations across 28 experiments), and cellular reactive oxygen species (CellROX™ Green) and mitochondrial mass/polarization (MitoTracker™ Deep Red) were quantified by fluorescence imaging. ASD fibroblasts displayed a hypermetabolic, uncoupled respiratory phenotype (~73% higher baseline respiration; ~109% higher proton leak; reduced coupling efficiency). Linear mixed models with polynomial dose terms revealed significant ASD × Cunermuspir complex interactions (ASD × Cunermuspir and/or their higher-order interactions with DMNQ and treatment) for four respiratory parameters. ASD cells exhibited lower steady-state oxidation-dependent CellROX™ Green fluorescence than controls despite greater respiratory uncoupling, as well as lower steady-state MitoTracker™ Deep Red fluorescence; Cunermuspir reshaped the MitoTracker™ DMNQ dose–response in an ASD-selective manner. These findings identify the Cunermuspir-associated modulation of the ASD mitochondrial and oxidant handling phenotype and motivate further mechanistic and translational evaluation.

## 1. Introduction

Copper is a redox-active essential transition metal whose cycling between the Cu(I) and Cu(II) oxidation states underlies electron transfer at the active centers of a defined set of cuproenzymes, most prominently cytochrome c oxidase (Complex IV; CcO) and the copper/zinc superoxide dismutases SOD1 (cytosolic and intermembrane space) and SOD3 (extracellular) [1,2]. The mitochondrial matrix superoxide dismutase SOD2 is manganese-dependent rather than copper-dependent and is therefore not a cuproenzyme. In the mitochondrion, copper is delivered through a dedicated intermembrane-space relay in which the metallochaperone COX17 hands Cu(I) to the SCO1/SCO2 and COX11 assembly factors, which in turn load the CuA site on COX2 and the CuB site on COX1, with COA6 acting as a thiol–disulfide oxidoreductase that maintains SCO1/SCO2 in a copper-competent state [3,4,5]. Because these copper centers catalyze the four-electron reduction of molecular oxygen to water at the terminal step of oxidative phosphorylation, disruption of mitochondrial copper handling simultaneously perturbs respiration and cellular redox balance [1,3,4].

Copper’s therapeutic and toxicological profile is dominated by this same redox chemistry. At physiological concentrations, restoring intramitochondrial copper can rescue Complex IV assembly defects in models of SCO2 and COA6 deficiency, stimulate mitochondrial biogenesis, and normalize respiration [3,6,7]. In contrast, unbuffered copper participates in Fenton-like reactions (Cu^+^ + H_2_O_2_ → Cu^2+^ + OH^−^ + OH•) that generate hydroxyl radicals and drive lipid, protein, and DNA oxidation, and supraphysiological copper triggers cuproptosis via lipoylated tricarboxylic acid cycle proteins [8,9]. The mitochondrion is therefore both the principal site of physiological copper utilization and the primary organelle at risk when copper handling is dysregulated, making cellular copper status a central determinant of the redox environment.

Autism spectrum disorder (ASD) provides a compelling clinical context in which to interrogate this copper–redox axis. Mitochondrial dysfunction is one of the most consistently replicated biological findings in ASD: 30–50% of affected individuals demonstrate biomarkers of abnormal mitochondrial function and up to 80% show abnormal electron transport chain (ETC) activity in immune cells [10,11]. Among ETC abnormalities, elevated Complex IV activity has been reported in muscle, fibroblasts, buccal epithelium, brain tissue, and lymphoblastoid cell lines (LCLs) from individuals with ASD [11,12,13]. This elevated Complex IV activity is accompanied by elevated basal respiration, increased proton leak, respiratory chain uncoupling, and mitochondrial superoxide elevation, reflecting a dysregulated hypermetabolic state rather than a classical loss-of-function mitochondrial disease [12,13]. Complementary evidence from mitochondrial carrier biochemistry and genetics further links ASD to altered mitochondrial aspartate/glutamate carrier (AGC1) function and calcium homeostasis [14], and a recent randomized double-blind placebo-controlled cross-over trial of a mitochondrial cofactor supplement in children with ASD reported improvements in both mitochondrial activity and clinical function, consistent with the therapeutic tractability of the ASD mitochondrial phenotype [15]. Independent evidence from tooth matrix biomarkers further implicates altered prenatal and early postnatal zinc–copper metabolic cycling in the emergence of ASD [16].

Despite converging evidence that copper handling and Complex IV function are simultaneously perturbed in ASD, the direct effect of copper supplementation on ASD mitochondrial respiration and cellular oxidant handling has not been systematically tested. We hypothesized that (i) ASD fibroblasts would exhibit a hypermetabolic, uncoupled respiratory phenotype relative to typically developing controls; (ii) exogenous Cu(I) delivery via Cunermuspir [copper(I)–niacin] would differentially remodel respiration in ASD versus control fibroblasts; and (iii) this differential response would extend to cellular reactive oxygen species (ROS) and mitochondrial mass/polarization readouts under a graded oxidative challenge. To test these hypotheses, we combined Seahorse XF respirometry with quantitative fluorescence imaging of CellROX™ Green (an oxidation-activated cellular ROS probe [17]) and MitoTracker™ Deep Red FM (a Cy5 probe whose accumulation depends on the mitochondrial volume and membrane potential [18,19]) under a factorial matrix of Cunermuspir concentration, treatment duration, and DMNQ challenge and analyzed the resulting 2133 respirometry observations and matched imaging data with linear mixed models incorporating polynomial dose terms.

## 2. Materials and Methods

### 2.1. Participants and Ethical Approval

The protocols for obtaining the ASD fibroblasts were registered at ClinicalTrials.gov (NCT02000284 and NCT02003170) and approved by the Institutional Review Board of the University of Arkansas for Medical Sciences (Little Rock, AR, USA). The study was conducted in accordance with the Declaration of Helsinki. Written informed consent was obtained from a parent or legal guardian of every participant prior to enrollment. Participants were recruited from the Arkansas Children’s Hospital Autism Multispecialty Clinic directed by the senior author; ASD diagnosis was established using previously described standardized criteria [13].

### 2.2. Fibroblast Cell Lines and Culture

Dermal fibroblast lines were derived from 9 children with ASD (AMC 007, AMC 109, AMC 146, AMC 147, AMC 163, AMC 168, AMC 315, AMC 440, AMC 553) by skin punch biopsy, as previously described [13]. Ten age-appropriate, typically developing control lines (GM01651, GM01864, GM02036, GM02037, GM03348, GM07753, GM08299, GM08398, GM08399, GM23973) were obtained from the Coriell Institute for Medical Research (Camden, NJ, USA). AMC cells were maintained in Dulbecco’s Modified Eagle Medium (DMEM) supplemented with 15% fetal bovine serum, 4.5 g/L D-glucose, and 110 mg/L sodium pyruvate at 37 °C under 5% CO_2_. GM cell lines were maintained at 37 °C under 5% CO_2_ in Minimal Essential Media (MEM) or DMEM with 15% FCS and supplements as specified by Coriell.

### 2.3. Cunermuspir Pre-Treatment and Oxidative Challenge

Fibroblasts were treated with Cunermuspir [copper(I)–niacin; BioCopper1; MitoSynergy, Flagstaff, AZ, USA] at 0, 50, or 100 µM (concentrations refer to the Cunermuspir complex, not copper equivalents) for 1 h or 24 h at 37 °C under 5% CO_2_. Immediately prior to respirometry or imaging assay, cells were exposed for 1 h to the redox-cycling naphthoquinone 2,3-dimethoxy-1,4-naphthoquinone (DMNQ) at 0, 0.05, 0.1, 0.25, 1.0, 2.5, or 5.0 µM. DMNQ generates intracellular superoxide and hydrogen peroxide through mitochondrial redox cycling and served as a graded oxidative stressor. The concentration range spanned low-micromolar to mid-micromolar exposures, chosen to produce a graded redox challenge.

### 2.4. Seahorse XF Respirometry

Cells were seeded at 35,000 cells per well in Seahorse XF96 microplates 24 h prior to assay. The mitochondrial oxygen consumption rate (OCR) and extracellular acidification rate (ECAR) were measured on the Seahorse XF Pro Analyzer (Agilent Technologies, Santa Clara, CA, USA) using the Cell Mito Stress Test protocol. Post-assay Hoechst 33342 staining was used, and data were normalized to the cell number using the Seahorse XF Imaging and Normalization System, using an Agilent BioTek Cytation 5 Cell Imaging Multimode Reader (Agilent Technologies Inc., Santa Clara, CA, USA), to verify the final cell density per well. Nuclei counts did not differ systematically between ASD and control wells, confirming that the reported per-well OCR/ECAR values were not confounded by the differential cell number at the time of assay. Cells were sequentially exposed to oligomycin (ATP synthase inhibitor), carbonyl cyanide-p-trifluoromethoxyphenylhydrazone (FCCP; uncoupler), and rotenone/antimycin A (Complex I and Complex III inhibitors, respectively). Eight respiratory parameters were derived: baseline respiration (BR), ATP-linked respiration (ALR), proton leak respiration (PLR), maximal respiration (MR), reserve capacity (RC), coupling efficiency (CE), the basal OCR/ECAR ratio (BOE), and the maximal OCR/ECAR ratio (MOE). Vulnerability to oxidative stress was quantified using the Mitochondrial Oxidative Stress Test (MOST) [20], in which cells were exposed to the DMNQ concentration series immediately prior to Seahorse profiling. A total of 2214 raw observations were collected (1126 ASD and 1088 non-ASD across 29 experiments); pre-specified outlier screening (Section 2.7) excluded one experiment (81 observations), yielding the final analytic dataset of 2133 observations (1084 ASD and 1049 non-ASD across 28 experiments and 19 cell lines).

### 2.5. Fluorescence Imaging of Cellular ROS and Mitochondrial Mass

Imaging was performed on two matched ASD–control fibroblast pairs: AMC 315 vs. GM03348 and AMC 163 vs. GM08399. Cells were seeded into 96-well Cellvis P96-1.5P imaging plates (Cellvis, Mountain View, CA, USA) with 0 or 50 µM for a single 24 h pre-treatment, crossed with DMNQ 0, 0.5, 2.5, or 5 µM as the acute oxidative challenge. Mitochondrial mass and polarization were labeled in PBS with MitoTracker™ Deep Red FM (Invitrogen™; Thermo Fisher Scientific, Waltham, MA, USA) at 400 nM for 30 min and imaged in the Cy5 channel using an Agilent BioTek Cytation 5 Cell Imaging Multimode Reader. This concentration was selected for the endpoint imaging protocol on the basis of empirical titration in these fibroblast lines, which showed that 400 nM achieved the most reliable signal-to-noise ratio after washing the wells 3 times with PBS. Prior work in primary human skin fibroblasts characterized the loading and membrane potential response of MitoTracker™ Deep Red FM under comparable staining conditions [18]. MitoTracker™ Deep Red FM accumulation depends on both the mitochondrial volume and the membrane potential. The membrane potential dependence has been characterized specifically for the Deep Red FM chemistry (as distinct from MitoTracker Green or CMXrosamine [21]) in Desai et al. 2024 in primary human skin fibroblasts [18], with complementary primary neuron characterization in Buckman et al. 2001 [22] and manufacturer-supplied specifications in [19]. Cellular ROS were labeled with DNA-bound CellROX™ Green (Invitrogen™) at 5 µM for 30 min and imaged in the GFP channel. CellROX™ Green is weakly fluorescent in its reduced state and becomes brightly fluorescent upon oxidation by superoxide and hydroxyl radicals with DNA binding [17]. Nuclei were counterstained with Hoechst 33342 (4 µM). Images were acquired using identical illumination, exposure, and gain settings per plate and analyzed with the BioTek Gen5 Software v3.18 (Agilent Technologies). Cells were segmented on the Hoechst channel, and the mean per-cell fluorescence intensities in the Cy5 and GFP channels were extracted only from segmented nuclei-associated cell masks. Because the outcome variable was a per-cell mean, differences in field-of-view cell density did not enter the readout. Per-cell intensities were then normalized within each plate to the matched untreated (0 µM Cunermuspir, 0 µM DMNQ) wells to remove between-plate scaling, and the log-transformed normalized per-cell intensities (log-normalized Cy5 for MitoTracker™; log-normalized GFP for CellROX™) served as the outcome variables. As a qualitative density check, field-by-field visual inspection at each Cunermuspir × DMNQ condition did not suggest systematic density differences between ASD and control wells.

### 2.6. Fibroblast Model Rationale

Dermal fibroblasts are a widely validated peripheral model for mitochondrial dysfunction in neurodevelopmental disorders. They share the same nuclear genome as neurons, are obtainable from living participants by minimally invasive biopsy, and retain cell-autonomous mitochondrial phenotypes that recapitulate ETC abnormalities documented in postmortem brain and immune cells from individuals with ASD [12,13]. Fibroblasts also permit controlled experimental manipulation, including copper supplementation and graded DMNQ challenge, that is not possible in postmortem tissue.

### 2.7. Complex IV Enzymatic Activity Assay (Exploratory Sub-Study)

As an exploratory sub-study to examine whether the respirometry-inferred Complex IV pattern was recoverable at the level of cytochrome c oxidase enzymatic activity, Complex IV activity was measured spectrophotometrically in three replicates in one ASD and one age-matched typically developing control line drawn from the same panel used in the respirometry cohort. The Complex IV Human Specific Activity Microplate Assay Kit (ab109910, Abcam Inc., Waltham, MA, USA) was used, following the manufacturer’s protocol. Cells were treated with Cunermuspir at 0 or 50 µM for 24 h under the conditions described in Section 2.3 and then harvested and assayed for Complex IV activity using kinetic linear-range determinations of ferrocytochrome c oxidation, yielding rate constants expressed in min^−1^.

### 2.8. Statistical Analysis

For each of the eight respiratory parameters, a linear mixed model (LMM) was fit, with the fixed effects being ASD status (categorical: ASD vs. non-ASD), Cunermuspir concentration (continuous: 0, 50, 100 µM of the nominal Cunermuspir complex), treatment duration (categorical: 1 h vs. 24 h), DMNQ concentration (continuous: 0, 0.05, 0.1, 0.25, 1.0, 2.5, 5.0 µM), quadratic terms for Cunermuspir and DMNQ, and all two-, three-, and four-way interactions among these predictors. Crossed random effects included a random intercept for Experiment and a variance component for Cell Line (implemented via ‘groups’ and ‘vc_formula’ in Python version 3.14.7 ‘statsmodels’ MixedLM). One experiment (Experiment 26; cell lines AMC 109 and GM03348) was identified as a statistical outlier (experiment-level BLUP z-scores > 2.0 across 5 of 8 dependent variables) and was excluded, yielding a final sample of N = 2133 (1084 ASD; 1049 control) from 28 experiments and 19 cell lines. Fixed effect significance was assessed by Wald z-tests. Effect sizes were expressed as percentage group differences and Wald z-statistics, because the partial η^2^ is not directly applicable to mixed models. Model adequacy was assessed by residual plots, Q–Q plots, Durbin–Watson statistics, Cook’s distance, and variance inflation factors (all VIFs < 10). Intraclass correlation coefficients (ICCs) were computed for Experiment and Cell Line.

For each imaging assay, the dependent variable was the log-transformed normalized per-cell fluorescence intensity. Fixed effects were ASD status (categorical), Cunermuspir concentration (categorical: 0 vs. 50 µM), and DMNQ concentration, entered as a three-degree orthogonal polynomial (DMNQ.L [linear], DMNQ.Q [quadratic], DMNQ.C [cubic]), with all two- and three-way interactions. Because ASD status was represented by two independent line pairs sampled across up to four independent experimental runs (CellROX™: 2 experiments; MitoTracker™: 4 experiments), LMMs were fit by REML with a random intercept for Experiment and a variance component for Cell Line, thereby accounting for the within-plate correlation of per-cell measurements. Models were fit per assay (CellROX™ n = 192 cells and MitoTracker™ n = 264). To formally test whether ASD-associated responses differed between the two readouts, a joint LMM was fit to both assays simultaneously (n = 456), with all fixed-effect terms interacted with an Assay factor (MitoTracker™ vs. CellROX™), yielding tests of Assay × ASD, Assay × ASD × DMNQ, and Assay × ASD × Cunermuspir × DMNQ heterogeneity. Wald tests were used for individual fixed effects, and model-based estimated marginal means with simple contrasts of ASD (Y−N) at each Cunermuspir × DMNQ combination were computed from the fitted LMMs. As a sensitivity analysis, the same fixed effect specification was refit by ordinary least squares regression pooling per-cell rows across experiments (Section 3.5).

For the Complex IV enzymatic assay, a LMM with fixed effects of ASD status (categorical: ASD vs. control), Cunermuspir (categorical: 0 vs. 50 µM nominal Cunermuspir complex concentration), and their interaction was fit by REML using a random intercept for cell line to accommodate the paired 0-versus-50 µM measurements within each line. The primary hypothesis test was the ASD × Cunermuspir interaction (equivalent to a test that the 0→50 µM Complex IV activity change differed between ASD and control lines). Given n = 3 per group, Wald z-tests were complemented by two exact/near-exact procedures: (i) a cluster bootstrap of the interaction coefficient with 5000 resamples of cell lines stratified by group, yielding percentile, basic, and bias-corrected-and-accelerated (BCa) 95% confidence intervals, and (ii) an exact permutation test enumerating all 20 subject-level relabelings of ASD-versus-control assignment, producing an exact two-sided *p*-value.

## 3. Results

### 3.1. ASD Fibroblasts Exhibit a Hypermetabolic, Uncoupled Respiratory Phenotype

**Figure 1:**
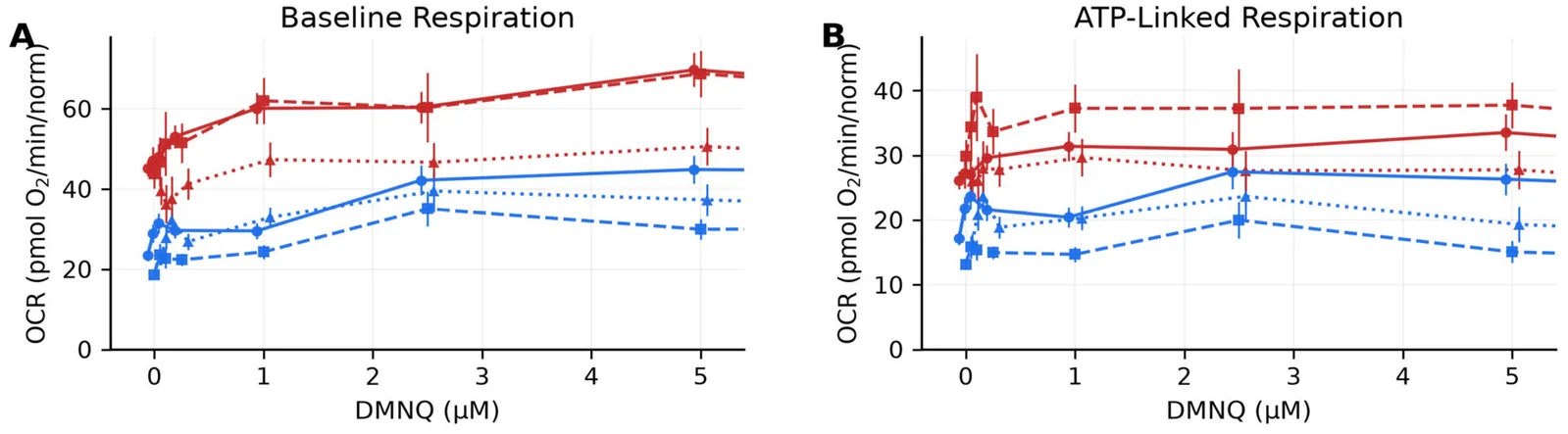

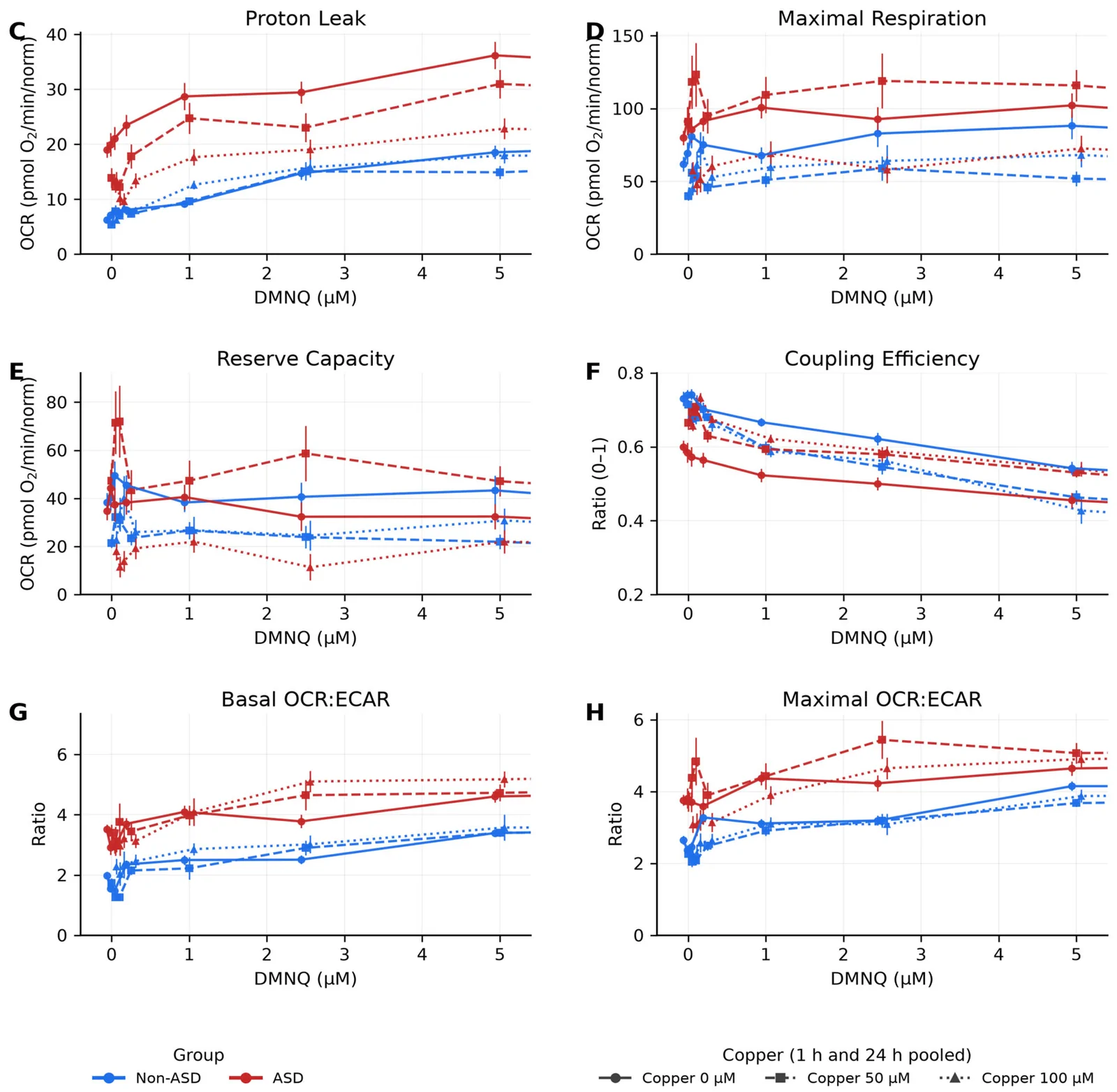
Combined respiratory phenotypes of ASD (red) and non-ASD (blue) fibroblasts across DMNQ concentrations and nominal Cunermuspir complex concentrations. Panels show all eight respiratory parameters: (**A**) baseline respiration (BR), (**B**) ATP-linked respiration (ALR), (**C**) proton leak (PLR), (**D**) maximal respiration (MR), (**E**) reserve capacity (RC), (**F**) coupling efficiency (CE), (**G**) basal OCR/ECAR ratio (BOE), and (**H**) maximal OCR/ECAR ratio (MOE). Within each panel, the nominal Cunermuspir complex concentration is encoded by the line style and marker shape (0 µM: solid/circle; 50 µM: dashed/square; 100 µM: dotted/triangle), pooling the 1 h and 24 h Cunermuspir exposures for compactness. Points are condition means; error bars are ±1 SEM across wells (small horizontal jitter added to separate overlapping Cunermuspir concentration levels). N = 2133 well-level observations from 28 experiments and 19 cell lines. ASD fibroblasts show elevated BR, PLR, MR, BOE, and MOE and reduced CE relative to non-ASD controls across DMNQ, with Cunermuspir reshaping several ASD dose–responses (statistical inferences in Table 1, Table 2 and Table 3).

All eight LMMs converged with non-zero variance for both random effects; 37–63% of the total variance in respiratory parameters was attributable to between-cell-line differences and 4–23% to between-experiment variation (Table 1). ASD status was a significant main effect for five of eight respiratory parameters (Table 2). ASD fibroblasts demonstrated significantly elevated BR (51.60 ± 0.96 vs. 29.89 ± 0.62; z = 2.59, *p* = 0.010; ~73% higher; Figure 1A). Both ALR (30.23 ± 0.63 vs. 19.66 ± 0.44; ~54% higher; Figure 1B) and MR (86.94 ± 2.08 vs. 63.27 ± 1.62; ~37% higher; Figure 1D) were numerically higher in ASD but did not reach statistical significance in the LMM. The most pronounced group difference was for PLR, which was ~109% higher in ASD than controls (21.37 ± 0.51 vs. 10.22 ± 0.23; z = 3.59, *p* < 0.001; Figure 1C), indicating substantial respiratory chain uncoupling. CE was correspondingly lower (0.59 ± 0.005 vs. 0.64 ± 0.005; z = −4.65, *p* < 0.001; Figure 1F). RC was 6% higher in ASD but not statistically significant (Figure 1E). Both BOE (3.77 ± 0.06 vs. 2.40 ± 0.05; z = 2.72, *p* = 0.007; Figure 1G) and MOE (4.03 ± 0.07 vs. 2.92 ± 0.04; z = 2.07, *p* = 0.039; Figure 1H) were significantly elevated in ASD, indicating a hypermetabolic, oxidative phosphorylation-biased phenotype.

### 3.2. DMNQ Induces Nonlinear Redox Dose-Dependent Changes

Both the linear and quadratic DMNQ terms were significant for most dependent variables (Table 3), confirming the nonlinear dose–response relationships between oxidative stress and mitochondrial function. The DMNQ linear and quadratic terms were significant for all parameters except RC, indicating inverted U or saturating dose–response profiles.

### 3.3. Cunermuspir Differentially Modulates Respiration in ASD Fibroblasts

ASD × Cunermuspir interactions were tested at both the linear and quadratic levels. The ASD × Cunermuspir (linear) interaction was significant for ALR (z = −2.38, *p* = 0.017); ASD × Cunermuspir^2^ was significant for ALR (z = 3.65, *p* < 0.001) and CE (z = 3.40, *p* < 0.001) (Table 3). Higher-order ASD × DMNQ interactions were significant for BR (ASD × DMNQ^2^ z = −2.54, *p* = 0.011) and PLR (ASD × DMNQ z = 3.31, *p* < 0.001 and ASD × DMNQ^2^ z = −4.04, *p* < 0.001). ASD × DMNQ and ASD × DMNQ^2^ were also significant for CE (z = 2.97, *p* = 0.003 and z = −2.61, *p* = 0.009, respectively). Considering the full ASD × Cunermuspir complex of terms (ASD × Cunermuspir, ASD × Cunermuspir^2^, ASD × Cunermuspir × Treatment, ASD × Cunermuspir^2^ × Treatment, and their higher-order interactions with DMNQ), nominally significant ASD-containing Cunermuspir complex terms were present for BR, ALR, PLR, and CE but not MR, RC, BOE, or MOE (Table 3).

### 3.4. Treatment Duration Modifies the Cunermuspir Response

Treatment duration (1 h vs. 24 h) exerted a significant main effect on CE (z = −2.65, *p* = 0.008). Treatment × DMNQ interactions were significant for ALR (z = 2.32, *p* = 0.020) and CE (z = 1.97, *p* = 0.049). Cunermuspir × Treatment interactions were significant for BOE (z = −2.99, *p* = 0.003) and MOE (z = −2.26, *p* = 0.024). Cunermuspir^2^ × Treatment interactions were significant for MR, RC, and BOE (Table 3). The three-way ASD × Cunermuspir × Treatment interaction was significant for ALR (z = 2.86, *p* = 0.004), PLR (z = −2.45, *p* = 0.014), and CE (z = 3.02, *p* = 0.003). The ASD × Cunermuspir^2^ × Treatment interaction was significant for ALR, PLR, and CE (Table 3). These three-way interactions indicate that the differential Cunermuspir response in ASD fibroblasts depends jointly on the Cunermuspir concentration and treatment duration.

### 3.5. ASD Fibroblasts Display Divergent Oxidation-Dependent CellROX™ and MitoTracker™ Responses (24 h Cunermuspir)

**Figure 2:**
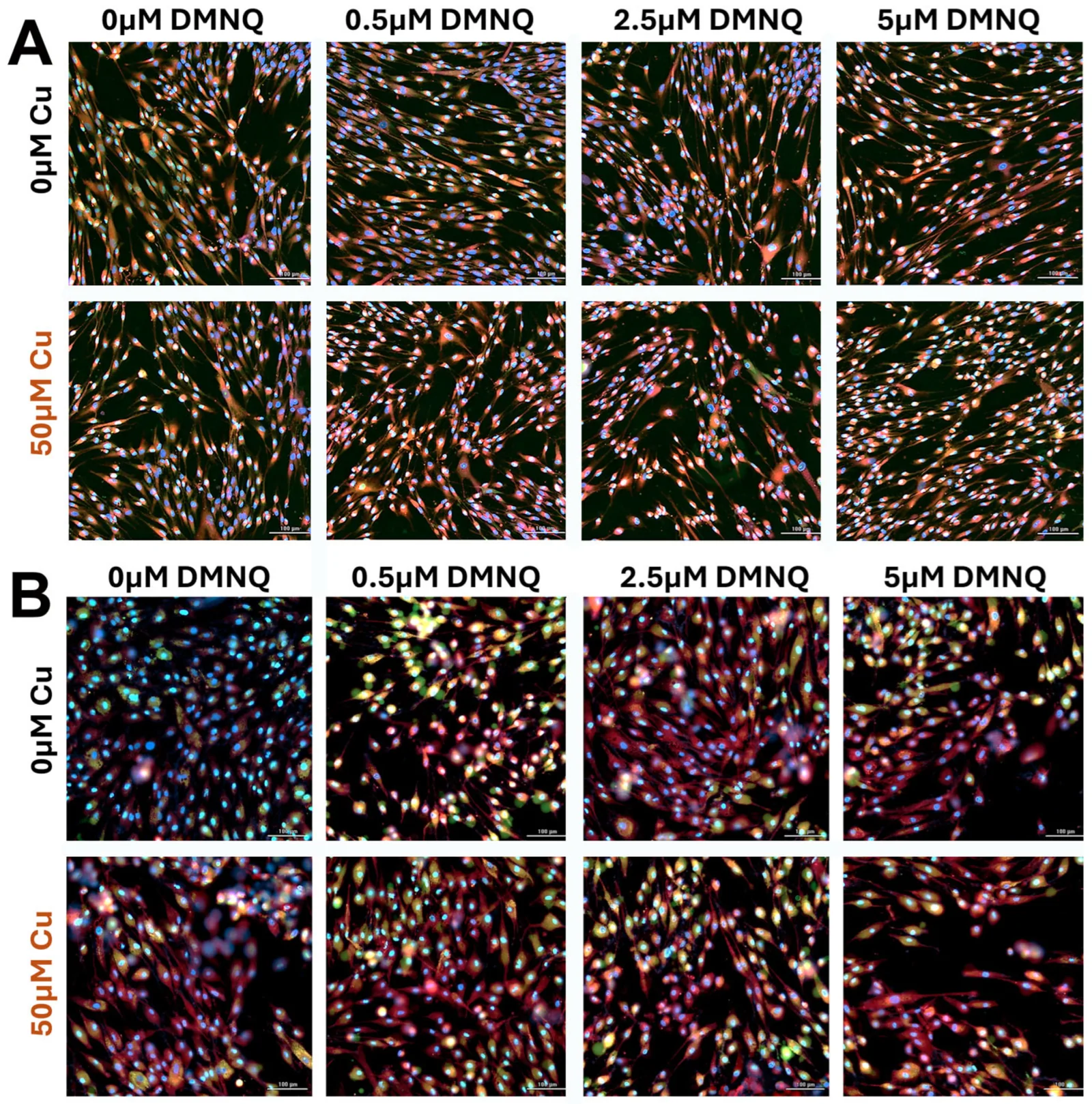
Representative photomicrographs from the AMC 315/GM03348 fluorescence imaging cohort: GM03348 ((**A**) control) and AMC 315 ((**B**) ASD) fibroblasts treated with 0 or 50 µM Cunermuspir and 0, 0.5, 2.5, or 5 µM DMNQ. Cells were stained with MitoTracker™ Deep Red FM, CellROX™ Green, and Hoechst 33342 as described in Section 2.5. Images were acquired using a 40× objective (scale bar = 100 µm). Statistical analyses (Section 3.5, Table 4) additionally included a second matched ASD–control pair (AMC 163/GM08399); representative images from this pair are available on request.

**Figure 3:**
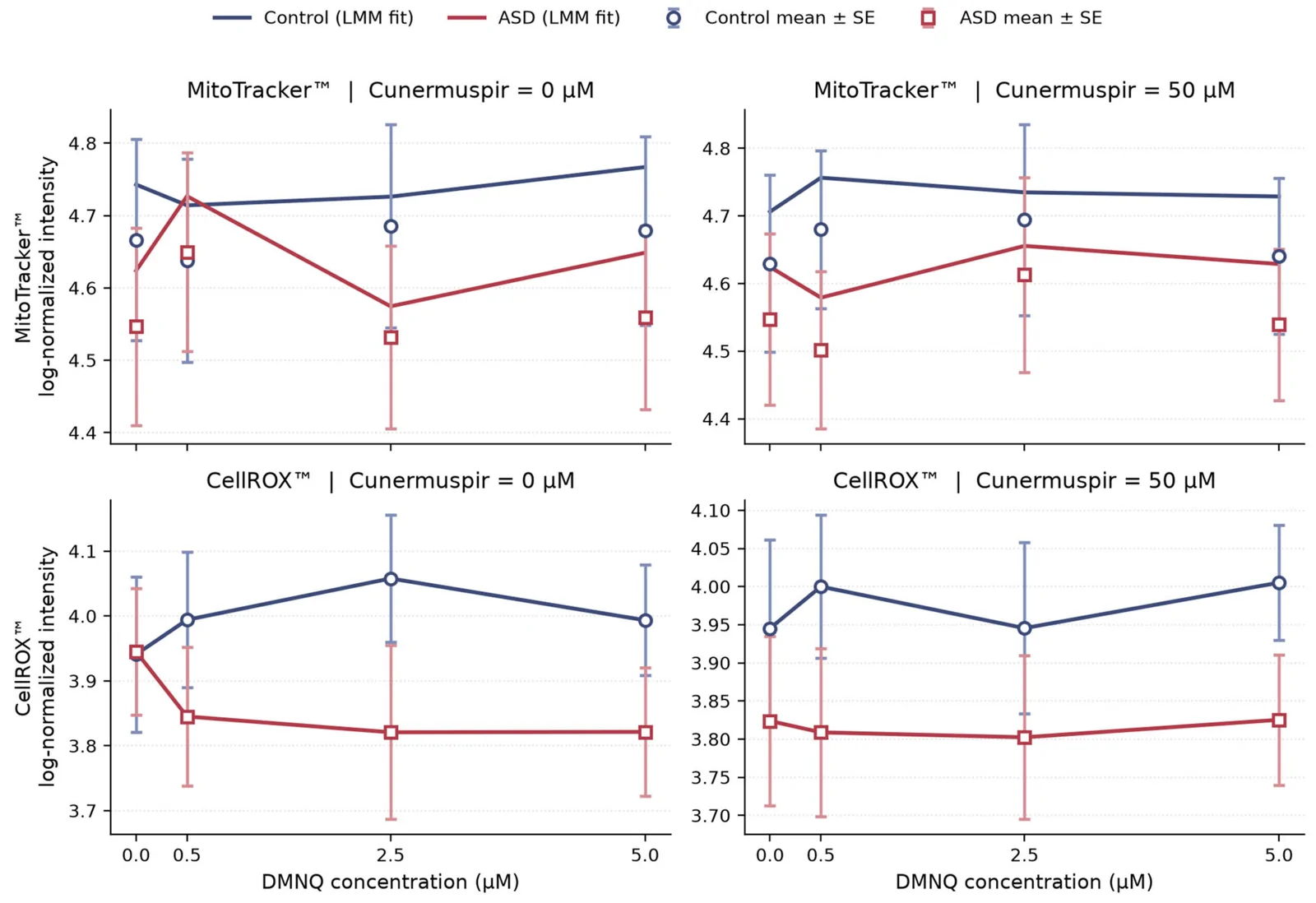
Pooled MitoTracker™ and CellROX™ dose–response across two independent matched ASD–control fibroblasts. Log-normalized fluorescence intensity (*y*-axis) is plotted against DMNQ concentration (*x*-axis); ASD lines are shown in red and controls in blue. Data are grouped by assay type (MitoTracker™ or CellROX™) and nominal Cunermuspir concentration (0 or 50 µM). Each point represents the group mean per condition; error bars are ± 1 SE across cells within conditions, and lines show fitted third-degree orthogonal polynomial dose–response curves from the pooled linear mixed model (Section 2.8). ASD-related differences are most apparent in the CellROX™ assay (highly significant ASD main effect and ASD × DMNQ.Q interaction), while, for MitoTracker™, they comprise a significant ASD main effect together with significant linear and higher-order ASD × Cunermuspir × DMNQ interactions.

Two independent matched ASD–control fibroblast pairs were imaged under a reduced Cunermuspir × DMNQ factorial (0 and 50 µM Cunermuspir; 0, 0.5, 2.5, and 5 µM DMNQ) relative to the Seahorse experiments (Figure 2 and Figure 3). LMMs with a random intercept for Experiment and a variance component for Cell Line were fit to the per-cell log-normalized fluorescence intensities. For CellROX™, a highly significant main effect of ASD status was found, with ASD fibroblasts exhibiting lower cellular ROS signals than controls (β = −0.186, SE = 0.038, z = −4.88, *p* = 1.1 × 10^−6^; Table 4). A significant DMNQ.Q response and an ASD × DMNQ.Q interaction (β = +0.102, z = 2.50, *p* = 0.012) indicated that ASD cells failed to mount the rising, convex CellROX™ dose–response seen in controls. Cunermuspir demonstrated a significant main effect (β = −0.072, *p* = 0.047). ASD CellROX™ intensities were lower across nearly all non-zero DMNQ doses, with statistically significant ASD-versus-control contrasts at 0.5 µM DMNQ (Y−N = −0.149, *p* = 0.0023 at 0 µM Cunermuspir; Y−N = −0.191, *p* = 1.0 × 10^−4^ at 50 µM Cunermuspir), 2.5 µM DMNQ (Y−N = −0.237, *p* = 8.6 × 10^−6^ at 0 µM Cunermuspir; Y−N = −0.143, *p* = 0.0072 at 50 µM Cunermuspir), and 5 µM DMNQ (Y−N = −0.172, *p* = 1.5 × 10^−4^ at 0 µM Cunermuspir; Y−N = −0.180, *p* = 7.7 × 10^−5^ at 50 µM Cunermuspir). In the MitoTracker™ dataset, the LMM revealed a significant main effect of ASD (β = −0.141, z = −3.75, *p* = 0.0002) together with highly significant ASD × DMNQ.L (z = −2.72, *p* = 0.0065), ASD × DMNQ.C (z = 2.45, *p* = 0.014), ASD × Cunermuspir × DMNQ.L (z = 3.16, *p* = 0.0016), and ASD × Cunermuspir × DMNQ.C (z = −2.92, *p* = 0.0035) interactions, indicating that ASD and control cells differed both in the overall level and in the copper-modulated shape of the MitoTracker™ dose–response.

A joint LMM combining both assays with all fixed effects interacting with Assay confirmed the significant heterogeneity of the ASD response between assays for the DMNQ cubic term (Assay × ASD × DMNQ.C z = 2.37, *p* = 0.018), with linear-order heterogeneity (Assay × ASD × DMNQ.L, *p* = 0.058). Between-experiment variability was substantial in both assays (0.20 for CellROX™; 0.39 for MitoTracker™), whereas between-line variability within groups was minimal after accounting for Experiment, indicating consistent ASD-related biology across both line pairs. Across matched conditions, the MitoTracker™−CellROX™ intensity gap remained substantially larger in ASD than controls at non-zero DMNQ, consistent with a coordinated divergence in which ASD cells show lower steady-state MitoTracker™ Deep Red fluorescence (β = −0.141, *p* = 0.0002) with the copper-dependent reshaping of the dose–response, alongside lower steady-state CellROX™ Green fluorescence (oxidation-dependent, signal).

### 3.6. Exploratory Complex IV Enzymatic Activity Sub-Study

**Figure 4:**
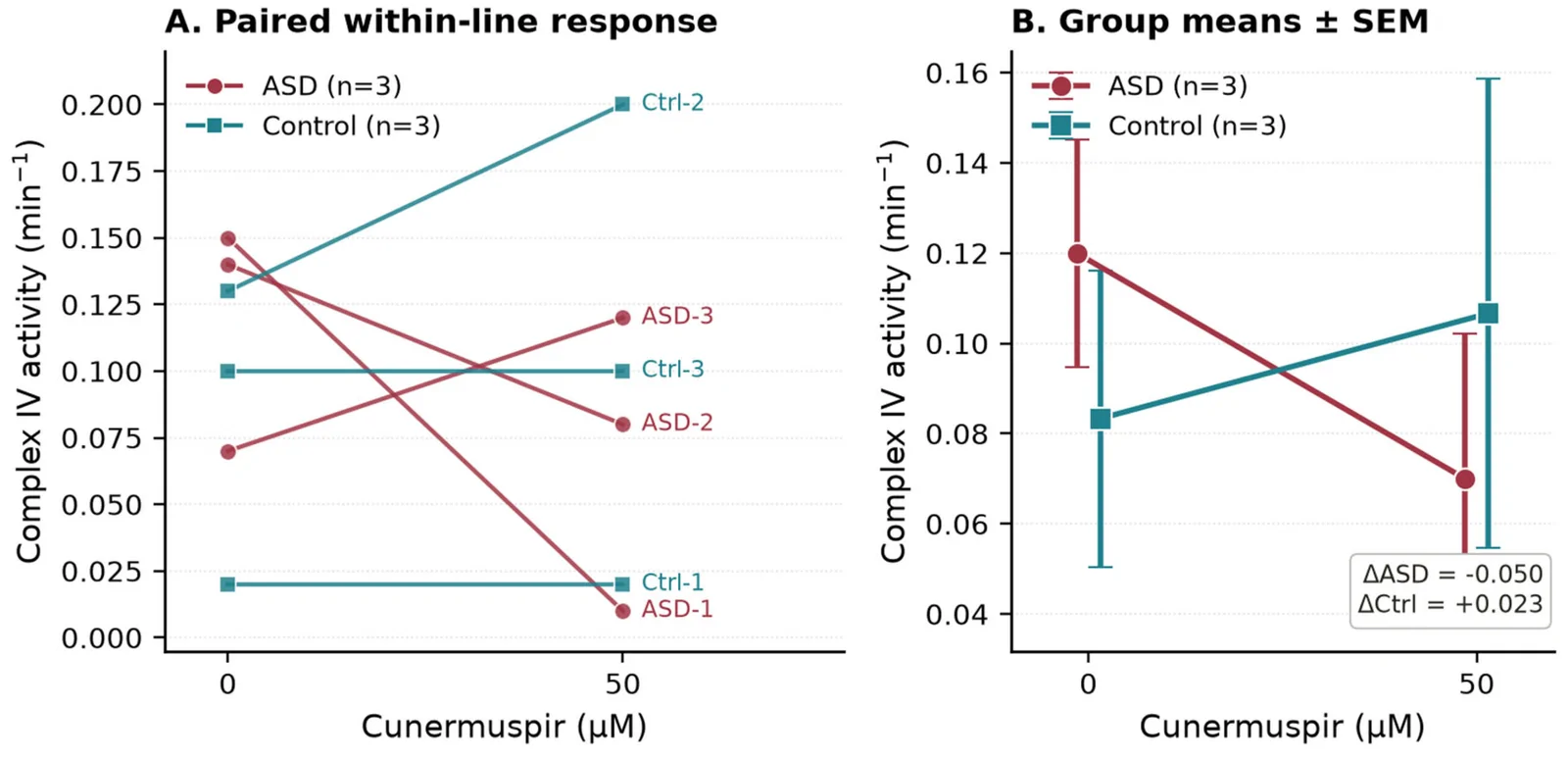
Direct spectrophotometric measurement of cytochrome c oxidase (Complex IV) enzymatic activity in a paired 0 vs. 50 µM Cunermuspir design across 3 ASD (red) and 3 non-ASD control (teal) fibroblast replicates drawn from the panel described in Section 2.2. (**A**) Paired within-line response: each trace shows the within-line 0→50 µM change; two of the three ASD replicates exhibited a within-line decrease (subject-level Δ: −0.14 and −0.06 min^−1^), while one ASD line increased (+0.05 min^−1^); two of the three control replicates were essentially unchanged. (**B**) Group means ± SEM at each dose. Baseline Complex IV activity was numerically higher in ASD lines (0.120 vs. 0.083 min^−1^). Under 50 µM Cunermuspir, mean ASD activity decreased by 0.050 min^−1^, whereas mean control activity was essentially unchanged (+0.023 min^−1^). The raw group-mean contrast was β = −0.073 min^−1^. The linear mixed-effects Wald test gave *p* = 0.220, cluster-bootstrap BCa 95% CI [−0.163, +0.013], and exact permutation *p* = 0.30 across all 20 subject-level relabelings.

As an exploratory sub-study not powered to test the ASD × Cunermuspir interaction, cytochrome c oxidase enzymatic activity was measured spectrophotometrically in a paired 0 vs. 50 µM Cunermuspir design across ASD and control fibroblast lines drawn from the panel described in Section 2.2 (Figure 4). Baseline (0 µM Cunermuspir) Complex IV activity was numerically higher in ASD than control lines (mean rate constant 0.120 vs. 0.083 min^−1^), directionally consistent with the elevated basal respiration and elevated OCR/ECAR ratios documented in the respirometry cohort (Section 3.1). Under 50 µM Cunermuspir, ASD showed a within-group decrease in Complex IV activity in two (Δ: −0.14, −0.06; +0.05) replicates, yielding a mean ASD change of −0.050 min^−1^. Control cell lines remained essentially unchanged (mean Δ = +0.023 min^−1^; subject-level Δ: 0.00, +0.07, 0.00). The raw group-mean contrast was β = −0.073 min^−1^. The corresponding linear mixed-effects estimate with a random intercept for Cell Line yielded |β| = 0.073 (bootstrap SE 0.049), with a parametric Wald *p* = 0.220, cluster-bootstrap BCa 95% CI [−0.163, +0.013] (two-sided bootstrap *p* = 0.139), and exact permutation *p* = 0.30 across all 20 subject-level relabelings. The direction and magnitude of the point estimate indicate a trend for Cunermuspir to selectively attenuate the elevated Complex IV activity of ASD fibroblasts, in the same direction as the respirometry-derived Cunermuspir × ASD interactions on ALR and CE (Section 3.3), but the result was not statistically significant in this small cohort.

## 4. Discussion

This study directly interrogates the copper–redox axis in ASD fibroblasts by combining respirometry with orthogonal fluorescence readouts of mitochondrial mass/polarization and cellular oxidant handling under a graded redox challenge. Three principal findings emerge. First, ASD fibroblasts display a hypermetabolic, uncoupled respiratory phenotype with elevated BR, PLR, and OCR/ECAR ratios and reduced CE, which is consistent with prior reports of Complex IV overactivation and respiratory chain uncoupling in ASD tissues [10,11,12,13]. Second, Cunermuspir differentially remodels this phenotype: significant ASD × Cunermuspir complex interactions were present for four respiratory parameters: BR, ALR, PLR, and CE (Table 3). Third, ASD fibroblasts paradoxically exhibit lower steady-state oxidation-dependent CellROX™ Green fluorescence than controls despite greater respiratory uncoupling, and Cunermuspir reshapes the MitoTracker™ DMNQ dose–response in an ASD-selective manner (significant ASD × Cunermuspir × DMNQ.L and DMNQ.C for MitoTracker™). Because no ionic copper, niacin-only, chelation, uptake, intracellular copper, or Cu speciation controls were included, we describe these findings as Cunermuspir-associated rather than definitively copper-mediated.

**Figure 5:**
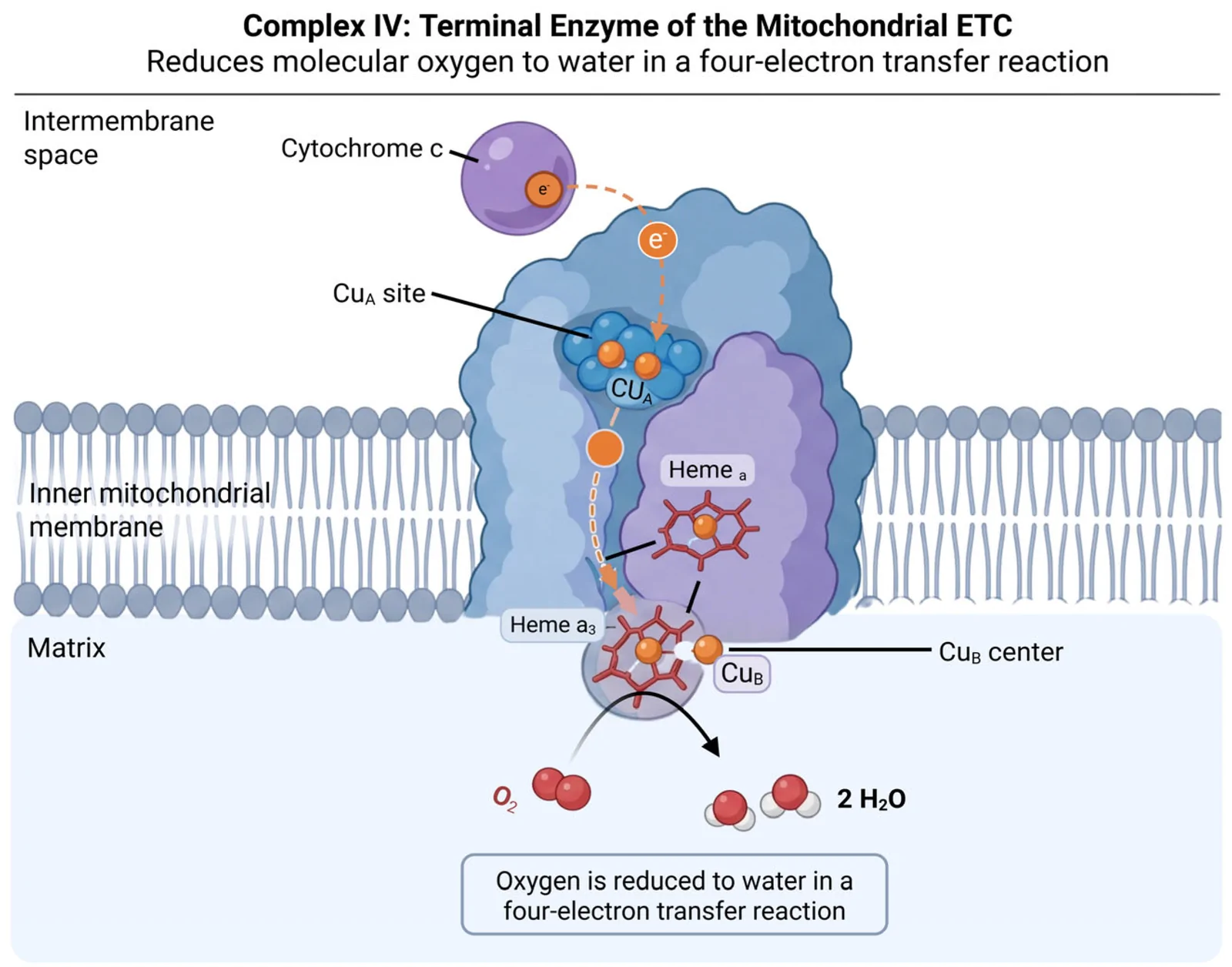
The role of copper in Complex IV (cytochrome c oxidase) function. Complex IV is the terminal enzyme of the mitochondrial electron transport chain and contains two copper centers (Cu_A_ and Cu_B_) and two heme groups. The Cu_A_ site accepts electrons from cytochrome c and transfers them to heme a and then to the Cu_B_–heme a_3_ binuclear active site, where molecular oxygen is reduced to water. Created in BioRender. Frye, R. (2026) https://BioRender.com/3bqrv03.

One plausible candidate mechanism involves Complex IV. Complex IV is the terminal cuproenzyme of the ETC and contains two redox-active copper centers (CuA, CuB) whose coordinated electron transfers reduce molecular oxygen to water [3,4] (Figure 5). Elevated Complex IV activity, driving increased BR and greater PLR, is a consistently replicated feature of ASD tissues [11,12,13]. Physiological Cu(I) delivery through the CTR1 → COX17 → SCO/COX11 relay is required to sustain Complex IV assembly and turnover, and copper supplementation rescues CcO assembly defects in SCO2 and COA6 disease models [3,6]. Our observation that Cunermuspir modulates ASD respiratory parameters in a nonlinear, treatment duration-dependent manner (significant ASD × Cunermuspir^2^ × Treatment interactions for ALR, PLR, and CE) is compatible with an action on Complex IV assembly and turnover, but the present design does not include the CTR1 uptake, intracellular copper quantification, or Complex IV assembly intermediate readouts that would be required to demonstrate such a mechanism. The 1 h versus 24 h contrast pooled across parameters does not parametrically resolve a minimum exposure time; time-course experiments with additional intermediate durations are needed.

An alternative but non-exclusive interpretation invokes compartmental copper insufficiency. Even with adequate systemic copper, defects at any step of the mitochondrial copper relay (CTR1, COX17, SCO1/SCO2, COX11, COA6) can produce functional mitochondrial copper deficiency [1,3,6]. Exogenous Cunermuspir could, in principle, compensate for such bottlenecks, but the present study did not measure CTR1-dependent uptake, intracellular copper accumulation, or Complex IV assembly intermediates and therefore cannot demonstrate either the uptake route or the biochemical mechanism. Direct experimental testing of the compartmental copper insufficiency hypothesis in ASD fibroblasts, including immunodetection of the mitochondrial copper relay proteins (CTR1, COX17, SCO1/SCO2, COA6) and compartmental copper quantification, is a logical next step.

A small exploratory spectrophotometric Complex IV sub-study (Section 3.6) was undertaken as a directional check on the respirometry pattern. Baseline (0 µM Cunermuspir) activity was numerically higher in ASD than control lines (0.120 vs. 0.083 min^−1^), and, under 50 µM Cunermuspir, the mean within-line change was numerically lower in ASD (Δ = −0.050 min^−1^) than in controls (Δ = +0.023 min^−1^). Although the ASD × Cunermuspir interaction did not reach statistical significance, the direction of the numerical difference is consistent with the Cunermuspir × ASD interactions on ALR and CE observed in the respirometry cohort (Section 3.3).

Copper’s redox chemistry cuts both ways. Cu(I)/Cu(II) cycling underlies both physiological oxygen reduction at Complex IV and the deleterious Fenton-like generation of hydroxyl radicals at unbuffered concentrations [8]. Supraphysiological copper causes mitochondrial cristae remodeling and cuproptosis via lipoylated TCA cycle proteins [8,9]. Brain mitochondria are particularly sensitive to copper overload [23]. The Cu(I)–niacin architecture of Cunermuspir provides a stabilized reduced-state donor. One working hypothesis is that Cunermuspir enters cells through the canonical high-affinity mammalian Cu(I) transporter CTR1 [24,25]; however, no direct experimental evidence for the CTR1-mediated uptake of Cunermuspir has been reported, this study did not measure it, and alternative uptake routes (passive diffusion of the intact complex, other divalent metal transporters, or endocytic entry) cannot be excluded. Direct experimental testing of the uptake route remains a priority for future biochemical work. Whether this physiological uptake route confers any therapeutic window advantage relative to the ionophoric membrane-permeable bypass used by elesclomol [26,27] cannot be determined from the present data, and no ionic Cu^2+^/Cu^+^, niacin-only, chelation, or elesclomol comparator arms were included in this study. The present experiments were also not designed to characterize Cunermuspir toxicity: overt viability or membrane integrity endpoints were not assayed, and the absence of a formal cytotoxicity readout precludes any conclusion about safety at the concentrations used. Dose optimization and independent replication in neuronal models remain a priority. Because dermal fibroblasts do not fully recapitulate neuronal cell type-specific redox biology, translational inferences to central nervous system copper pharmacology should be regarded as hypothesis-generating pending validation in neural cells and in vivo systems.

The fluorescence imaging results add a redox-specific layer to the respirometric phenotype. The lower steady-state CellROX™ signal in ASD despite greater proton leak was unexpected, because elevated basal respiration and uncoupling are classically associated with increased mitochondrial superoxide production [10,12]. Several non-exclusive mechanisms are consistent with the data. First, ASD fibroblasts may upregulate cytosolic and mitochondrial antioxidant systems (notably glutathione and thioredoxin, both known to be perturbed in ASD [10,11]) to a degree that offsets ROS generated by the leaky ETC, producing lower steady-state CellROX™ Green fluorescence (oxidation-dependent, signal). Second, proton leak itself is mildly cytoprotective: partial uncoupling depolarizes the inner membrane and reduces single-electron escape from the ETC, decreasing superoxide flux. Third, CellROX™ Green reports a specific pool of oxidized species and signals [17] and may undersample compartmentalized mitochondrial ROS scavenged before reaching the probe. The trend-level ASD × Cunermuspir × DMNQ.Q interaction for CellROX™ (z = −1.86, *p* = 0.063) is suggestive that the relationship between extracellular redox cycling stress and the CellROX™-detectable oxidant pool may itself be remodeled by copper, but this requires confirmation in an adequately powered replication. The MitoTracker™ result of a significant ASD main effect combined with significant linear and higher-order ASD × Cunermuspir × DMNQ interactions indicates that ASD fibroblasts differ from controls both in the overall level and in the copper-modulated shape of the mitochondrial mass/polarization dose–response, consistent with active network remodeling (biogenesis, fission/fusion, membrane potential homeostasis) under graded oxidative stress and paralleling previously reported morphological changes in ASD fibroblasts [13]. Importantly, this fluorescence imaging signature was observed in a design that combined two independent matched ASD–control fibroblast pairs, and the linear mixed model attributed essentially none of the residual variance to between-line differences within groups after accounting for the experimental variance, indicating that the ASD-associated CellROX™ and MitoTracker™ phenotypes are consistent across the two independently ascertained line pairs.

Taken together, the respirometric and imaging findings sketch a coordinated ASD phenotype in which the oxidation-dependent CellROX™ Green signal and steady-state MitoTracker™ Deep Red signal are both lower than in matched controls (β = −0.141, *p* = 0.0002 for MitoTracker™) against a background of elevated basal respiration and pronounced proton leak. Cunermuspir retunes rather than uniformly reverses these axes, with significant ASD-selective effects for a subset of respiratory and imaging endpoints. Because no ionic copper, niacin-only, chelation, or Cu speciation controls were included, the mechanistic attribution of these effects to copper per se, as opposed to Cunermuspir-associated actions, cannot be established from these data.

Several limitations warrant emphasis. The exploratory spectrophotometric Complex IV sub-study (Section 3.6) was not powered to test the ASD × Cunermuspir interaction and was a directional check only. The Complex IV assay itself provides an integrated activity readout and does not resolve the contributions of holoenzyme assembly, cofactor loading, and catalytic turnover. Seahorse parameters similarly reflect integrated ETC output. Copper may modulate respiration through mitochondrial biogenesis, ROS signaling to ETC gene expression, membrane potential effects that are independent of Complex IV, and glycolytic reprogramming, in addition to direct Complex IV assembly effects [3,4,5].

The fibroblast model, while validated for ASD mitochondrial studies [12,13], does not fully capture neural cell type-specific redox biology, and translational inferences to central nervous system copper handling must therefore be regarded as hypothesis-generating rather than conclusive. The Cunermuspir complex concentration range used here (0–100 µM of the intact complex) refers to nominal Cunermuspir complex concentrations delivered to the culture medium and is not equivalent to copper-equivalent or free copper concentrations; because the free copper fraction under these culture conditions depends on ligand exchange with serum proteins and other media components and was not measured, a direct quantitative comparison with the physiological free copper concentrations in plasma or brain interstitial fluid is not supported by the present data. The concentrations used here are within the range routinely applied in in vitro Cu complex exposure studies. Unlike whole plasma, culture media do not contain ceruloplasmin or the full complement of high-affinity Cu(I)/Cu(II) binding proteins that buffer free copper in vivo, and, even with 15% fetal bovine serum, the extracellular Cu-buffering capacity of the culture environment is not equivalent to that of plasma. The concentrations used here were therefore not intended to model in vivo free Cu exposure. Any translational extrapolation therefore requires deliberate in vitro–in vivo dose conversion and benchmarking against published animal copper supplementation models and clinical Cu speciation data before dosing decisions can be made [9,23].

DMNQ likewise does not replicate the full complexity of in vivo oxidative stress. The present design also did not include a copper chelation arm (e.g., bathocuproine disulfonate or tetrathiomolybdate); pairing copper loading with orthogonal copper withdrawal in future work would allow the direct causal attribution of the observed redox and respiratory shifts to bioavailable copper, as opposed to niacin-associated or off-target ligand effects, and would complement head-to-head comparisons of Cunermuspir with ionic Cu^2+^/Cu^+^ salts, niacin-only controls, and the copper ionophore elesclomol [26,27].

The fluorescence imaging analysis still draws on a small number of ascertained ASD and control donors; further replication in larger, independently ascertained ASD and control cohorts is required. The imaging panel also relied on the general cellular ROS probe CellROX™ Green and the mass/polarization-sensitive probe MitoTracker™ Deep Red FM; it did not include a mitochondria-selective superoxide indicator such as MitoSOX Red or the direct quantification of glutathione (reduced and oxidized, GSH/GSSG) or thioredoxin pathway proteins, which would be required to distinguish cytosolic from mitochondrial ROS pools and to test the hypothesis that ASD fibroblasts upregulate antioxidant capacity as a compensatory response.

The direct compartmental quantification of copper (cytosolic vs. mitochondrial) and immunodetection of the mitochondrial copper relay proteins (CTR1, COX17, SCO1/SCO2, COA6) were outside the scope of this initial phenotyping study and are a logical next step to test the compartmental copper insufficiency hypothesis directly. The imaging arm used a single 24 h Cunermuspir pre-treatment, so no claim about the duration dependence of the imaging response can be derived from these data. Critically, the present study did not include an ionic copper arm (Cu^2+^ or Cu^+^ salts), a niacin-only arm, a copper chelation arm (e.g., BCS or tetrathiomolybdate), a Cu uptake blockade arm, or direct measurement of intracellular or mitochondrial copper or copper speciation. All effects are therefore Cunermuspir-associated rather than definitively copper-mediated, and the specific claims of CTR1-dependent uptake, restored Complex IV assembly, a wider therapeutic window, and the absence of copper toxicity are not supported by the present data. Future studies should therefore incorporate MitoSOX and GSH/GSSG assays, compartmental copper measurements, mitochondrial DNA copy number quantification, membrane potential measurements, targeted analysis and immunodetection of the copper relay proteins (CTR1, COX17, SCO1/SCO2, COA6), and a head-to-head comparison of Cunermuspir with ionic copper salts, niacin-only controls, copper chelators, and copper ionophores such as elesclomol [26,27] to define the safest and most efficient delivery strategy.

## 5. Conclusions

In this in vitro fibroblast study, Cunermuspir differentially modulated mitochondrial respiration and cellular oxidant handling between ASD and control donors. ASD fibroblasts showed a hypermetabolic, uncoupled respiratory phenotype paired with lower steady-state oxidation-dependent CellROX™ Green fluorescence and lower steady-state MitoTracker™ Deep Red fluorescence (β = −0.141, *p* = 0.0002). Nominally significant ASD × Cunermuspir complex interactions were present for four respiratory parameters (BR, ALR, PLR, and CE), and Cunermuspir reshaped the MitoTracker™ DMNQ dose–response in an ASD-selective manner. Because no ionic Cu, niacin-only, chelation, uptake, intracellular copper, or Cu speciation controls were included, these effects are described as Cunermuspir-associated rather than definitively copper-mediated, and we do not draw conclusions about CTR1-dependent uptake, Complex IV assembly, therapeutic windows, or Cunermuspir toxicity. Translation to Cunermuspir- or copper-based strategies for ASD would require independent replication in neuronal and in vivo models, direct compartmental copper and copper chaperone measurements, mitochondria-selective ROS and antioxidant pathway readouts (MitoSOX, GSH/GSSG, thioredoxin), and rigorous in vitro to in vivo dose conversion.

## Figures and Tables

**Table 1:** Model summary for linear mixed model analyses of eight respiratory parameters. N = 2133 for all models. Models include crossed random effects for Experiment (random intercept) and Cell Line (variance component). Log-Lik = log-likelihood; AIC = Akaike information criterion; Var = variance component; ICC = intraclass correlation coefficient.

Parameter	N	Log-Lik	AIC	Var (Exp)	Var (Cell)	Var (Resid)	ICC (Exp)	ICC (Cell)
BR	2133	−8813.4	17,704.7	145.41	317.70	179.19	0.226	0.495
ALR	2133	−7930.4	15,938.8	57.52	168.50	76.92	0.190	0.556
PLR	2133	−7494.2	15,066.3	23.60	75.19	51.26	0.157	0.501
MR	2133	−10,568.4	21,214.8	572.96	1998.90	953.96	0.163	0.567
RC	2133	−9900.9	19,879.8	139.37	1098.58	505.69	0.080	0.630
CE	2133	1480.8	−2883.6	0.00	0.01	0.01	0.110	0.379
BOE	2133	−3748.5	7575.0	0.55	1.17	1.46	0.172	0.369
MOE	2133	−3726.3	7530.5	0.13	1.66	1.42	0.040	0.516

**Table 2:** ASD group differences in respiratory parameters. Means ± SE are shown for ASD (n = 1084) and non-ASD control (n = 1049) fibroblasts. z-statistics and *p*-values from Wald tests of the ASD main effect in the linear mixed model.

Parameter	ASD Mean ± SE	Control Mean ± SE	% Diff	z	*p*
BR	51.60 ± 0.96	29.89 ± 0.62	73%	2.59	0.010
ALR	30.23 ± 0.63	19.66 ± 0.44	54%	1.16	0.248
PLR	21.37 ± 0.51	10.22 ± 0.23	109%	3.59	3.34 × 10^−4^
MR	86.94 ± 2.08	63.27 ± 1.62	37%	1.23	0.217
RC	35.34 ± 1.42	33.38 ± 1.12	6%	0.27	0.790
CE	0.59 ± 0.005	0.64 ± 0.005	−9%	−4.65	3.35 × 10^−6^
BOE	3.77 ± 0.06	2.40 ± 0.05	57%	2.72	0.007
MOE	4.03 ± 0.07	2.92 ± 0.04	38%	2.07	0.039

**Table 3:** Significant effects matrix. z-values are shown for all significant effects across eight respiratory parameters from linear mixed models with crossed random effects. Significance: * *p* < 0.05, ** *p* < 0.01, *** *p* < 0.001. Only effects that were significant for at least one dependent variable are shown. Blank cells indicate non-significant effects.

Effect	BR	ALR	PLR	MR	RC	CE	BOE	MOE
ASD	2.59 **		3.59 ***			−4.65 ***	2.72 **	2.07 *
Treatment						−2.65 **		
ASD/Cunermuspir		−2.38 *						
Cunermuspir/Treatment							−2.99 **	−2.26 *
ASD/Cunermuspir/Treatment		2.86 **	−2.45 *			3.02 **		
Cunermuspir^2^						−2.47 *		
ASD/Cunermuspir^2^		3.65 ***				3.40 ***		
Cunermuspir^2^/Treatment				−2.88 **	−3.65 ***		3.48 ***	
ASD/Cunermuspir^2^/Treatment		−3.27 **	2.41 *			−2.58 **		
DMNQ	7.34 ***	4.29 ***	8.47 ***	3.37 ***		−12.24 ***	4.87 ***	5.16 ***
ASD/DMNQ			3.31 ***			2.97 **		
Treatment/DMNQ		2.32 *				1.97 *		
ASD/Treatment/DMNQ						−2.04 *		
DMNQ^2^	−5.84 ***	−4.32 ***	−5.62 ***	−3.61 ***		7.78 ***	−3.76 ***	−4.08 ***
ASD/DMNQ^2^	−2.54 *		−4.04 ***			−2.61 **		
Cunermuspir/DMNQ	−2.96 **	−2.90 **	−1.99 *	−1.98 *		−2.51 *		
ASD/Cunermuspir/DMNQ	2.45 *	2.77 **						
Cunermuspir/DMNQ^2^	2.66 **	2.45 *	1.97 *					
ASD/Cunermuspir/DMNQ^2^	−2.12 *	−2.28 *						
Cunermuspir/Treatment/DMNQ^2^						2.01 *		−2.06 *
Cunermuspir^2^/DMNQ	3.30 ***	3.07 **	2.41 *	2.35 *				
ASD/Cunermuspir^2^/DMNQ	−3.15 **	−3.28 **						
Cunermuspir^2^/DMNQ^2^	−3.07 **	−2.60 **	−2.57 *	−2.29 *				
ASD/Cunermuspir^2^/DMNQ^2^	2.81 **	2.64 **	2.02 *					
Cunermuspir^2^/Treatment/DMNQ^2^								2.11 *

**Table 4:** Fluorescence imaging statistical results. Wald z-statistics and *p*-values from per-assay linear mixed-effects models (LMMs) for MitoTracker™ (MT) and CellROX™ (CR), each with a random intercept for Experiment and a variance component for Cell Line, and from the joint two-assay LMM in which each fixed-effect term was interacted with Assay (the joint columns report the z-statistic and *p*-value for the Assay × Effect interaction, i.e., whether the effect differs between MT and CR). DMNQ is encoded as orthogonal polynomial contrasts (.L = linear, .Q = quadratic, .C = cubic). Significance: * *p* < 0.05, ** *p* < 0.01, *** *p* < 0.001. Only terms relevant to ASD/Cunermuspir/DMNQ effects are shown.

Effect	z (MT)	*p* (MT)	z (CR)	*p* (CR)	z (Joint)	*p* (Joint)
ASD	−3.75	<0.001 ***	−4.88	<0.001 ***	0.78	0.434
Cunermuspir	−0.19	0.848	−1.99	0.047 *	1.17	0.242
DMNQ.L	0.79	0.428	0.09	0.927	0.41	0.682
DMNQ.Q	0.69	0.488	−2.10	0.036 *	1.79	0.074
DMNQ.C	−0.50	0.616	0.35	0.723	−0.54	0.592
ASD × Cunermuspir	1.17	0.241	0.79	0.431	0.22	0.829
ASD × DMNQ.L	−2.72	0.007 **	0.31	0.758	−1.89	0.058
ASD × DMNQ.Q	0.56	0.577	2.50	0.012 *	−1.26	0.207
ASD × DMNQ.C	2.45	0.014 *	−1.31	0.190	2.37	0.018 *
ASD × Cunermuspir × DMNQ.L	3.16	0.002 **	0.37	0.714	1.72	0.086
ASD × Cunermuspir × DMNQ.Q	−0.53	0.593	−1.86	0.063	0.87	0.385
ASD × Cunermuspir × DMNQ.C	−2.92	0.004 **	0.16	0.873	−1.91	0.056

## Data Availability

The de-identified data supporting the findings of this study, together with the analysis code, are available from the corresponding author upon reasonable request, subject to any restrictions imposed by the approving institutional review board and applicable privacy regulations.

## Abbreviations

ALR, ATP-linked respiration; ASD, autism spectrum disorder; BLUP, best linear unbiased predictor; BOE, basal OCR/ECAR ratio; BR, baseline respiration; CcO, cytochrome c oxidase; CE, coupling efficiency; COA6, cytochrome c oxidase assembly factor 6; CTR1, high-affinity copper transporter 1; DMEM, Dulbecco’s Modified Eagle Medium; DMNQ, 2,3-dimethoxy-1,4-naphthoquinone; ECAR, extracellular acidification rate; ETC, electron transport chain; FCCP, carbonyl cyanide-p-trifluoromethoxyphenylhydrazone; ICC, intraclass correlation coefficient; LCL, lymphoblastoid cell line; LMM, linear mixed model; MOE, maximal OCR/ECAR ratio; MOST, Mitochondrial Oxidative Stress Test; MR, maximal respiration; OCR, oxygen consumption rate; PBS, phosphate-buffered saline; PLR, proton leak respiration; RC, reserve capacity; ROS, reactive oxygen species; SCO1/SCO2, synthesis of cytochrome c oxidase 1/2; SOD1, copper/zinc superoxide dismutase 1 (cytosolic and intermembrane space); SOD2, manganese superoxide dismutase 2 (mitochondrial matrix; not a cuproenzyme); SOD3, copper/zinc superoxide dismutase 3 (extracellular); VIF, variance inflation factor.

## References

[1] Cobine P.A., Moore S.A., Leary S.C. (2021). Getting Out What You Put In: Copper in Mitochondria and Its Impacts on Human Disease. Biochim. Biophys. Acta Mol. Cell Res..

[2] Ruiz L.M., Libedinsky A., Elorza A.A. (2021). Role of Copper on Mitochondrial Function and Metabolism. Front. Mol. Biosci..

[3] Ghosh A., Trivedi P.P., Timbalia S.A., Griffin A.T., Rahn J.J., Chan S.S.L., Gohil V.M. (2014). Copper Supplementation Restores Cytochrome c Oxidase Assembly Defect in a Mitochondrial Disease Model of COA6 Deficiency. Hum. Mol. Genet..

[4] Horn D., Barrientos A. (2008). Mitochondrial Copper Metabolism and Delivery to Cytochrome c Oxidase. IUBMB Life.

[5] Garza N.M., Swaminathan A.B., Maremanda K.P., Zulkifli M., Gohil V.M. (2023). Mitochondrial Copper in Human Genetic Disorders. Trends Endocrinol. Metab..

[6] Jett K.A., Leary S.C. (2018). Building the CuA Site of Cytochrome c Oxidase: A Complicated, Redox-Dependent Process Driven by a Surprisingly Large Complement of Accessory Proteins. J. Biol. Chem..

[7] Barrientos A., Barros M.H., Valnot I., Rötig A., Rustin P., Tzagoloff A. (2002). Cytochrome Oxidase in Health and Disease. Gene.

[8] Tsvetkov P., Coy S., Petrova B., Dreishpoon M., Verma A., Abdusamad M., Rossen J., Joesch-Cohen L., Humeidi R., Spangler R.D. (2022). Copper Induces Cell Death by Targeting Lipoylated TCA Cycle Proteins. Science.

[9] Behzadfar L., Abdollahi M., Sabzevari O., Hosseini R., Salimi A., Naserzadeh P., Sharifzadeh M., Pourahmad J. (2017). Potentiating Role of Copper on Spatial Memory Deficit Induced by Beta Amyloid and Evaluation of Mitochondrial Function Markers in the Hippocampus of Rats. Metallomics.

[10] Rossignol D.A., Frye R.E. (2012). Mitochondrial Dysfunction in Autism Spectrum Disorders: A Systematic Review and Meta-Analysis. Mol. Psychiatry.

[11] Frye R.E., Rincon N., McCarty P.J., Brister D., Scheck A.C., Rossignol D.A. (2024). Biomarkers of Mitochondrial Dysfunction in Autism Spectrum Disorder: A Systematic Review and Meta-Analysis. Neurobiol. Dis..

[12] Rose S., Frye R.E., Slattery J., Wynne R., Tippett M., Pavliv O., Melnyk S., James S.J. (2014). Oxidative Stress Induces Mitochondrial Dysfunction in a Subset of Autism Lymphoblastoid Cell Lines in a Well-Matched Case Control Cohort. PLoS ONE.

[13] Frye R.E., Lionnard L., Singh I., Karim M.A., Chajra H., Frechet M., Kissa K., Racine V., Ammanamanchi A., McCarty P.J. (2021). Mitochondrial Morphology Is Associated with Respiratory Chain Uncoupling in Autism Spectrum Disorder. Transl. Psychiatry.

[14] Palmieri L., Papaleo V., Porcelli V., Scarcia P., Gaita L., Sacco R., Hager J., Rousseau F., Curatolo P., Persico A.M. (2010). Altered Calcium Homeostasis in Autism-Spectrum Disorders: Evidence from Biochemical and Genetic Studies of the Mitochondrial Aspartate/Glutamate Carrier AGC1. Mol. Psychiatry.

[15] Hill Z., McCarty P.J., Boles R.G., Frye R.E. (2025). A Mitochondrial Supplement Improves Function and Mitochondrial Activity in Autism: A Double-Blind Placebo-Controlled Cross-Over Trial. Int. J. Mol. Sci..

[16] Curtin P., Austin C., Curtin A., Gennings C., Arora M., Tammimies K., Willfors C., Berggren S., Siper P., Rai D. (2018). Dynamical Features in Fetal and Postnatal Zinc–Copper Metabolic Cycles Predict the Emergence of Autism Spectrum Disorder. Sci. Adv..

[17] Thermo Fisher Scientific CellROX™ Green Reagent, for Oxidative Stress Detection (Catalog No. C10444): Product Information and Technical Reference. https://www.thermofisher.com/order/catalog/product/C10444.

[18] Desai S., Grefte S., van de Westerlo E., Lauwen S., Paters A., Prehn J.H.M., Gan Z., Keijer J., Adjobo-Hermans M.J.W., Koopman W.J.H. (2024). Performance of TMRM and Mitotrackers in Mitochondrial Morphofunctional Analysis of Primary Human Skin Fibroblasts. Biochim. Biophys. Acta Bioenerg..

[19] Thermo Fisher Scientific MitoTracker™ Deep Red FM (Catalog No. M22426): Product Information and Technical Reference. https://www.thermofisher.com/order/catalog/product/M22426.

[20] Frye R.E., McCarty P.J., Werner B.A., Rose S., Scheck A.C. (2024). Bioenergetic Signatures of Neurodevelopmental Regression. Front. Physiol..

[21] Pendergrass W., Wolf N., Poot M. (2004). Efficacy of MitoTracker Green and CMXrosamine to Measure Changes in Mitochondrial Membrane Potentials in Living Cells and Tissues. Cytometry.

[22] Buckman J.F., Hernandez H., Kress G.J., Votyakova T.V., Pal S., Reynolds I.J. (2001). MitoTracker Labeling in Primary Neuronal and Astrocytic Cultures: Influence of Mitochondrial Membrane Potential and Oxidants. J. Neurosci. Methods.

[23] Borchard S., Bork F., Rieder T., Hao C., Woitalla D., Bürkle A., Zischka H. (2018). The Exceptional Sensitivity of Brain Mitochondria to Copper. Toxicol. Vitr..

[24] Kuo Y.M., Gybina A.A., Pyatskowit J.W., Gitschier J., Prohaska J.R. (2006). Copper Transport Protein (Ctr1) Levels in Mice Are Tissue Specific and Dependent on Copper Status. J. Nutr..

[25] Öhrvik H., Thiele D.J. (2015). The Role of Ctr1 and Ctr2 in Mammalian Copper Homeostasis and Platinum-Based Chemotherapy. J. Trace Elem. Med. Biol..

[26] Soma S., Latimer A.J., Chun H., Vicary A.C., Timbalia S.A., Boulet A., Rahn J.J., Chan S.S.L., Leary S.C., Kim B.E. (2018). Elesclomol Restores Mitochondrial Function in Genetic Models of Copper Deficiency. Proc. Natl. Acad. Sci. USA.

[27] Zulkifli M., Spelbring A.N., Zhang Y., Gohil V.M. (2023). FDX1-Dependent and Independent Mechanisms of Elesclomol-Mediated Intracellular Copper Delivery. Proc. Natl. Acad. Sci. USA.

